# Analysis of temporal virus evolution and intra-host diversity in long-term non-progressors by bulk next-generation sequencing

**DOI:** 10.1128/spectrum.02227-25

**Published:** 2026-03-30

**Authors:** Rongfeng Chen, Jianwen Mo, Yuanting Li, Yuting Wu, Feirong Chen, Qinglian Qin, Hong Yang, Changping Xie, Kang Li, Yuyuan Huang, Wudi Wei, Zongxiang Yuan, Li Ye, Hao Liang, Junjun Jiang

**Affiliations:** 1 Guangxi Key Laboratory of AIDS Prevention and Treatment & School of Public Health, Guangxi Medical University 654463, Nanning, Guangxi, China; 2 Joint Laboratory for Emerging Infectious Diseases in China (Guangxi)-ASEAN, Life Sciences Institute, Guangxi Medical University 666892, Nanning, Guangxi, China; 3 Lingshan Center for Disease Control and Prevention, Qinzhou, Guangxi, China; 4 Liuzhou Center for Disease Control and Prevention 651534 , Liuzhou, Guangxi, China; 5 School of Basic Medical Sciences, Guangxi Medical University 74626, Nanning, Guangxi, China; University of Miami, Miami, Florida, USA

**Keywords:** HIV-1, long-term non-progressors, next-generation sequencing, viral evolution, intra-host diversity, cytotoxic T lymphocyte epitopes

## Abstract

Long-term non-progressors (LTNPs) provide key insights into HIV-1 pathogenesis and potential functional cure strategies. Whether viral evolution persists in LTNPs remains debated. Previous studies have mainly focused on partial HIV-1 genome fragments using single-gene amplification. In contrast, our study employs near full-length bulk next-generation sequencing (NGS) to investigate viral quasispecies evolution, diversity, and CTL-associated mutations at the nucleotide level, offering new perspectives on intra-host viral dynamics. Blood samples of two LTNPs were collected at four time points over a approximately 2 years. The HIV-1 near full-length genome of plasma RNA and proviral DNA were amplified, and bulk NGS was performed. Consensus sequences were used for phylogenetic analysis and divergence calculation. Intra-host single-nucleotide variants (iSNVs), amino acid variations, and mutations in CTL epitopes were also analyzed. Both LTNPs displayed limited evolution over time, as identified from the phylogenetic tree and genetic distance, with higher divergence observed in DNA sequences than in RNA sequences. Evolutionary divergence was observed between HIV-1 plasma RNA and proviral DNA sequences. Intra-host diversity indices, including the number of iSNVs and Shannon entropy, also indicated greater diversity in DNA sequences than in RNA sequences. A considerable number and high frequency of mutations in CTL were identified consistently both in DNA and in RNA sequences, which showed slight frequency changes over time. These findings support the limit HIV-1 evolution in LTNPs and greater intra-host diversity of proviral DNA than plasma RNA. Our findings also suggest substantial, high-frequency, and sustained mutations in CTL epitopes in LTNPs with a relatively high level of viral replication despite optimal immune control.

**IMPORTANCE:**

Long-term non-progressors (LTNPs) are a rare group of HIV-1-infected individuals who maintain stable CD4^+^ T cell counts and low viral loads in the absence of antiretroviral therapy. Understanding the virological and immunological mechanisms underpinning this phenotype could inform strategies for functional cure. Using bulk next-generation sequencing of near full-length genomes of both HIV plasma RNA and proviral DNA over multiple time points, our study provides high-resolution insights into intra-host viral evolution in LTNPs. We reveal limited viral evolution and divergence between plasma RNA and proviral DNA. Higher diversity was found in proviral DNA than in plasma RNA, contributed by a substantial number of low-frequency mutations. HLA-restricted CTL escape mutations were stable and maintained at high frequency across all time points. These results challenge the notion of viral stasis in LTNPs and underscore the complex interplay between viral persistence and immune control, offering critical clues for vaccine design and reservoir-targeting therapeutic approaches. These findings support the hypothesis that LTNPs maintain durable viral control and non-progressive disease by achieving a balance between viral evolution and immune containment.

## INTRODUCTION

Human immunodeficiency virus type 1 infection (HIV-1) is characterized by persistent viral replication and the decline of CD4^+^ T cells, leading to impairment of the immune system and increasing the risk of opportunistic infections and malignant tumors. Currently, multiple antiviral therapies are potent in virus suppression. However, lifelong medication is required for HIV-1 infections, as discontinuation typically results in rapid viral rebound within several weeks. Therefore, antiretroviral therapy (ART)-free control of HIV replication is a major objective of functional cure therapies. Among persons with human immunodeficiency virus (PWH), a unique subgroup known as long-term non-progressors (LTNPs) is characterized by the ability to maintain normal CD4^+^ T cell counts (>500 cells/µL) (1–3) and an asymptomatic state for 7–10 years without antiretroviral therapy (4), which provides a model very close to ART-free remission. Additionally, a subset of LTNPs, termed “elite controllers (EC),” can maintain an undetectable viral load (5). Several factors, including CCR5Δ32 deletion, protective HLA alleles such as HLA-B*57 and HLA-B*27, attenuated viral strains, and strong T cell activity, contribute to spontaneous control in LTNPs. Although some of these immune and genetic traits are rarely detected in the broader population, recognizing the immune and genetic traits of HIV controllers can help understand the complex interplay between genetic, immune, and virological factors. These immune and genetic traits can be mimicked or translated into therapies toward broader PLWH, ultimately achieving a functional cure.

One intense research topic is the characteristics of the HIV-1 genome in LTNPs, and whether viral evolution continues in LTNPs is under debate. Only <1% nucleotide variation was detected in near full-length HIV-1 proviruses derived from one non-progressor during a time span of 8 years (6). In a larger cohort of HIV-1 controllers, some controllers exhibited relatively diverse proviral populations and measurable divergence over 10 years of follow-up (7). Several studies also demonstrate the evidence of ongoing evolution in plasma viruses and PBMC reservoir by the analysis of *pol* gene or *env* gene (4, 8, 9). The inconsistent observations indicate high heterogeneity in viral quasispecies among HIV-1 controllers.

Due to the difficulty in preserving longitudinal specimens and PCR amplification of long segments, studies focused on the long-term evolution of HIV-1 controllers, generally based on partial HIV-1 genomic sequences instead of near full-length genome, such as *env*, *pol,* or *nef* genes. Moreover, the limited number of PCR products obtained from single-genome amplification is insufficient to reflect the intra-host diversity. Recent advances in NGS technologies and bioinformatics have significantly promoted research on viral evolution in HIV-1-infected individuals (10–12). Quantitative measures of within-host viral genetic diversity based on viral quasispecies sequencing can provide nucleotide aspects of diversity and have been done in many virus species (13, 14). Hence, systemic analysis of near-full length of plasma RNA and proviral DNA quasispecies derived from NGS can gain a better understanding of the genetic characteristics of LTNPs.

HLA class I alleles, which define the personal repertoire of targetable HIV CTL epitopes, are associated with viral control because they enable a more effective antiviral CD8^+^ T cell response (15). One promising direction in HIV vaccine design is to recapitulate the T-cell responses observed in natural controllers by targeting critical, conserved epitopes that are restricted by protective HLA alleles. Amino acid changes present in central, terminal, or flanking regions of CTL epitopes impair the effectiveness of the immune response. It is hypothesized that the reservoir in these patients could be enriched in wild-type sequences of epitopes recognized by CTL cells. However, multiple CD8 escape mutations are detected in proviral DNA from HIV-1 controllers (16, 17). Whether CD8 escape mutations are concurrently present in plasma RNA and blood proviral DNA, and the dynamics of the emergence of mutations over time, are not fully understood.

In this study, we investigated the evolutionary and intra-host diversity in two LTNPs through longitudinal follow-up. By using bulk PCR and deep sequencing of near full-genome genomes, evolution and intra-host diversity of plasma viral RNA and simultaneous PBMC-derived proviral DNA were analyzed. Moreover, antibody-associated variations in the *env* gene and mutations in CTL epitopes were also identified.

## MATERIALS AND METHODS

### Study subjects

A cohort of LTNPs is established and followed up based on the following criteria: (i) inclusion criteria: infected with HIV-1 for at least 7 years, no history of ART, has at least five records of CD4^+^ T cell counts tests and maintaining CD4^+^ T cell counts >500 cells/µL for every test, and no AIDS-related clinical symptoms or diseases. (ii) Exclusion criteria: co-infection with hepatitis B, hepatitis C, or treponema pallidum; presence of serious chronic diseases, including cardiovascular disease, renal disease, and autoimmune disease; and inability to provide a written informed consent document. Participants were followed up every 6–12 months since diagnosis to perform infection-monitoring tests and collect demographics and clinical data.

**Fig 1:**
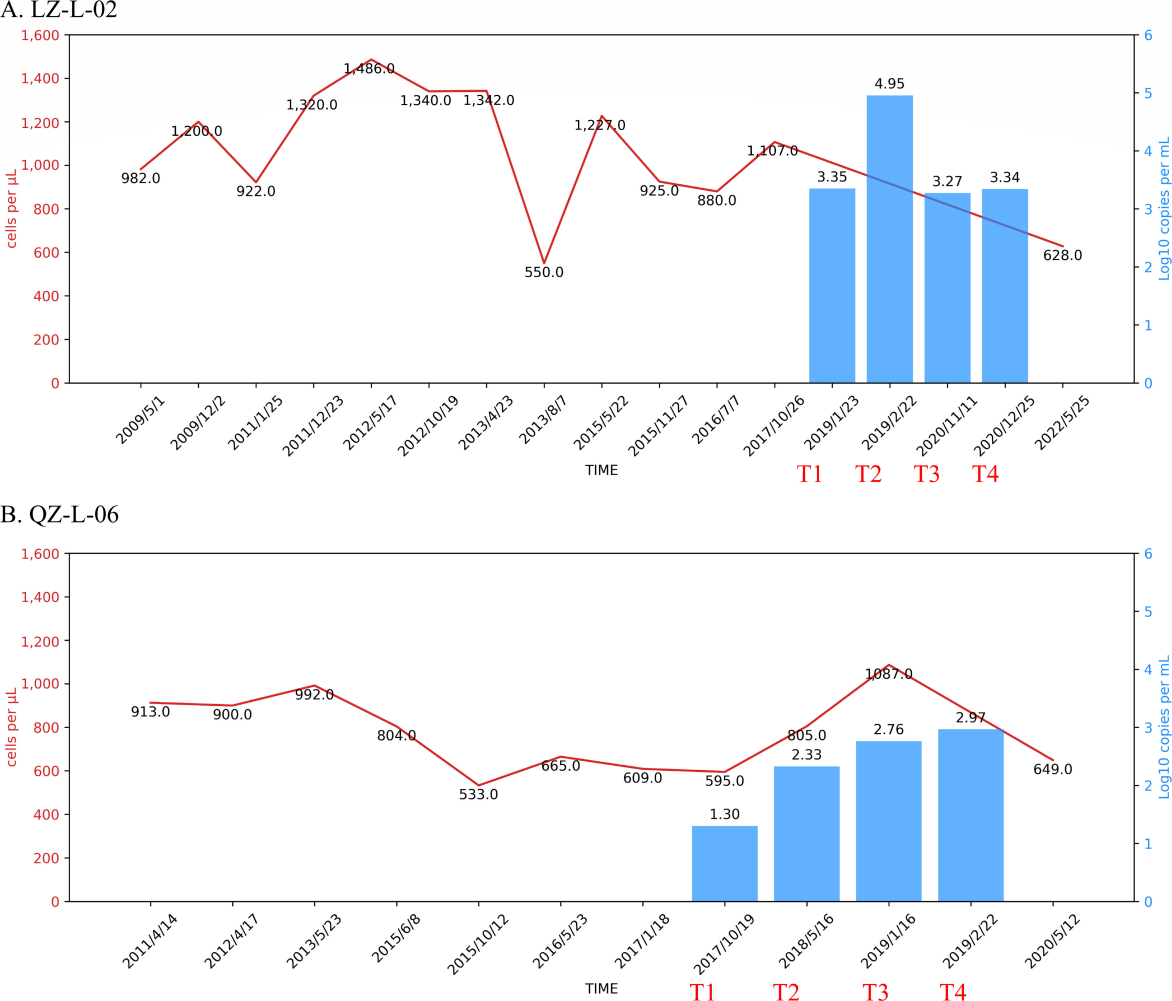
Longitudinal clinical follow-up of LZ-L-02 and QZ-L-06. CD4^+^ T cell counts (cells/µL) and plasma HIV-1 RNA viral load (log10 copies/mL) since HIV diagnosis are shown on the left and right y-axes, respectively. T1–T4 indicate the four follow-up time points with paired CD4^+^ T cell counts and HIV-1 RNA viral load data selected for analysis. (**A**) CD4^+^ T cell counts and plasma HIV-1 RNA viral load of LZ-L-02. (**B**) CD4^+^ T cell counts and plasma HIV-1 RNA viral load of QZ-L-06.

In this study, two LTNPs from the cohort, LZ-L-02 and QZ-L-06, were selected for analysis who were diagnosed with HIV-1 infection in 2007 and 1999, respectively. Blood samples of four time points during 2017 and 2020 were collected (Fig. 1).

### Blood sample collection, CD4^+^ T cell counts, and viral load testing

At each follow-up, 10 mL peripheral blood was collected in EDTA-anticoagulant tube and submitted to the Guangxi Key Laboratory of AIDS Prevention and Treatment within 6 h. Blood was separated into plasma and cells by Ficoll-Paque Plus density gradient centrifugation. Aliquots of plasma and PBMCs were stored at −80°C until use.

CD4^+^ T cell counts were performed in whole fresh blood on a FACS Calibur (Becton Dickinson, San Jose, CA, USA) using the TRITEST three-color CD4/CD8/CD3 reagent and TRUCOUNT tubes (Becton Dickinson). The HIV-1 viral load was determined by Xpert HIV-1 Viral Load (Xpert, Cepheid, Sunnyvale, California; limit of detection: 40 copies/mL) assay according to the manufacturer’s instructions.

### HLA class I genotyping and CCR5 genotyping

PCR-SBT (sequence-based genotyping) was used to identify Class I major histocompatibility complex (MHC I) alleles in two LTNPs. Briefly, target gene fragments were amplified using the QIAGEN LongRange PCR kit according to the manufacturer’s protocol. The amplification effect was confirmed using 1% agarose gel electrophoresis. The amplification products were purified according to the ExoI/SAP method, which should be completed within 24 h. After purification, sequencing of the HLA position was performed. The presence of CCR5Δ32 deletion was assessed by PCR amplification/agarose gel electrophoresis as previously described (18).

### Near full-length HIV-1 genome amplification and NGS

Near full-length HIV-1 genome amplification covering positions 1,023–9,024 according to the HXB2 coordinates was performed according to protocols described by Banin et al. (19) and Gall et al. (20). RNA was extracted using the QIAGEN QIAamp Viral RNA kit following the manufacturer’s protocol. The extracted RNA was then subjected to reverse transcription to synthesize cDNA. Nested PCR reactions were performed in a total reaction volume of 25 µL per sample, containing 11.5 µL nuclease-free water, 0.5 µL PrimeSTAR GXL DNA polymerase (Takara Bio), 5 µL 5× PrimeSTAR GXL buffer, 2.5 µL dNTP mix (2.5 mM each), 0.5 µL forward and reverse primers (10 μM), and 5 µL cDNA. The PCR reactions were performed with the following cycling conditions: initial denaturation step of 30 s at 98°C and 32 cycles of 10 s at 98°C, 15 s at 53°C, and elongation at 68°C for 30-60 seconds per 1 kb of the amplification product. After 32 cycles, a final elongation step was performed at 68°C for 5 min, and the mix was stored at 4°C. The amplification products were confirmed using 1% agarose gel electrophoresis.

DNA was extracted from 5 × 10⁶ PBMCs using the QIAamp DNeasy Blood and Tissue Kits (QIAGEN), following the manufacturer’s protocol, with a final elution volume of 30 µL. Nested PCR reactions were performed in a total reaction volume of 25 µL per sample containing 1.5 µL nuclease-free water, 12.5 µL 2X premix of TaKaRa Ex Premier DNA Polymerase, 0.5 µL forward and reverse primers, and 5 µL DNA. The PCR reactions were performed with the following cycling conditions: initial denaturation step of 1 min at 94°C and 38 cycles of 10 s at 98°C, 15 s at 58°C, and elongation at 68°C for 3.5 min. After 38 cycles, a final elongation step was performed at 68°C for 2 min, and the mix stored at 4°C. The amplification effect was confirmed using 1% agarose gel electrophoresis.

### Library preparation for next-generation sequencing

Amplicons of each sample were purified using AMPure XP beads (Beckman Coulter) and quantitated using the Qubit dsDNA HS Assay Kit (ThermoFisher) according to the manufacturer’s instructions. DNA libraries were prepared using the Hieff NGS MaxUp II DNA Library Prep Kit for Illumina (Yeasen) according to the manufacturer’s instructions. Libraries were purified by Hieff NGS DNA Selection Beads (Yeasen). The size of libraries was checked by electrophoresis on a 2% agarose gel, and the molarity was quantitated using the Qubit dsDNA HS Assay Kit (ThermoFisher). The pooled library was sequenced on a Novaseq 6000 Illumina platform via 2 × 150 bp paired-end sequencing using the 300 cycle v1.5 kit (Illumina, #20028312).

### NGS data analysis

SmaltAlign (21) software was used to align the reads with reference HXB2 (GenBank accession number K03455) and generate a consensus sequence containing mix-bases. A custom R script was applied to refine the consensus sequences by replacing mix-bases with the most frequent base in ambiguous positions. Haplotypes of each sample were reconstructed using CliqueSNV (22) for each sample (HXB2: 1023-9024).

### Phylogenetic analyses

BioEdit was used to align the sequences with HIV-1 subtype reference sequences. Maximum-likelihood (ML) phylogenetic trees were inferred from aligned sequences sets using MEGA7 following best-fitting models of nucleotide substitution calculation. The initial tree was constructed automatically (Default-NJ/BioNJ), and the tree topologies were searched using subtree-pruning-and-regrafting level 3 (SPR level 3). The confidence of each node in phylogenetic trees was determined using the bootstrap test with 1,000 replicates. A cluster with a bootstrap value above 0.7 was considered high reliability. Divergence for each subject was defined as the pairwise genetic distance between T1 and T2, T3, and T4, respectively. Pairwise genetic distances were estimated by the best-fitting model using MEGA7.

### Intra-host diversity

We used a variant calling file generated by SmaltAlign software, which contains single-nucleotide polymorphisms (SNPs) and their frequencies. Positions with a sequencing depth >1,000 were included for analysis, and mutations with a frequency >0.01 were identified as intra-host single-nucleotide variants (iSNVs). Position-wise Shannon entropy (*Hi*) was calculated for each iSNV (23). The mean Shannon entropy (*avgH*) for a gene *X* of length *N* was defined as the sum of *Hi* over all iSNVs within gene *X*, divided by *N*. The *avgH* for near full-length genome (HXB2: 1023-9024), *gag* (HXB2: 1023-2292), *pol* (HXB2: 2253-5096), and *env* (HXB2: 6225-8795) was calculated, respectively. The relative diversity of a gene was defined as its *avgH* relative to that of the near full-length genome. The software VirVarSeq (23) was used to calculate intra-host amino acid changes and frequencies of the three structural genes.

A sensitivity analysis was performed to assess the robustness of the intra-host diversity results. Specifically, we varied the mutation filtration thresholds, re-identifying iSNVs using stricter thresholds of >0.05 and >0.10, respectively.

### Characteristics of the variable region in the *env* gene

The *env* sequences were initially aligned using Gene Cutter, and then, amino acid sequences of V1 (aa 131–149), V2 (aa 158–197), V3 (aa 296–331), V4 (aa 385–418), and V5 (aa 460–471) were aligned with reference HXB2. The length of the variable region (V1–V5) and the number of potential N-linked glycosylation sites (PNGS) were analyzed using the online analysis software Variable Region Characteristics at the Los Alamos HIV database website. Additionally, amino acid sequences of contact regions for CD4-binding site (CD4bs) antibodies, including the inner domain, Loop D, and β23/loop V5/β24 along the HIV-1 gp120, were visualized with Jalview (version 2.11.1.4).

### Prediction of optimal CTL immune response epitopes and mutation analysis

The best-defined CTL/CD8^+^ epitopes were identified using the epitope list from the Los Alamos Immunology Database. The term “best-defined” refers to epitopes capable of efficiently activating CD8^+^ T cell responses at low concentrations (24). Mutations present within best-defined CTL/CD8^+^ epitopes were identified. A mutated epitope is considered a CTL escape epitope if it belongs to the “CTL/CD8+ Epitope Variants and Escape Mutations HIV database” according to the HLA class I genotype carried by subjects.

### Quantification of total HIV-1 DNA

Total HIV-1 DNA was amplified according to the method previously described (25), with modifications to the primers made in-house.

### Statistics analysis

To examine the effect of sampling time on genetic divergence, we initially performed simple linear regression for each sample, modeling genetic divergence as a function of time. This yielded the time-trend coefficient (β₁) along with its 95% CI, standard error, and *P* value. To control for multiple comparisons across the three independent tests, *P* values were adjusted using the Benjamini-Hochberg false discovery rate (FDR) procedure. Model fit was assessed using the coefficient of determination (R²). All analyses were conducted in R version 4.4.2, with statistical significance set at α = 0.05.

## RESULTS

### Participant characteristics

In this study, we included two LTNPs (LZ-L-02 and QZ-L-06) defined as subjects infected with HIV-1 for at least 10 years that maintained a normal range of CD4^+^ T cell counts (above 500 cells/µL) in every monitoring test without antiretroviral therapy. LZ-L-02 is a male and was diagnosed with HIV-1 infection in 2007. QZ-L-06 is a female and was diagnosed with HIV-1 infection in 1999. Both of the two LTNPs were intravenous drug users and have been followed up since diagnosis. QZ-L-06 was classified as viremia control because she displayed detectable viremia (51–2,000 copies/mL), but the viral load of LZ-L-02 was higher than 2,000 copies/mL for three time points (Fig. 1). Protective HLA class I alleles were detected in both LTNPs, which are HLA-B*58:01 and HLA-B*51:01, respectively (Table 1), whereas CCR5 delta32 deletion was not found in either of them. Blood samples collected from four time points for each LTNP were used for investigation, designated as T1, T2, T3, and T4, respectively (Fig. 1). For subject LZ-L-02, only plasma RNA sequences from four time points were obtained through HIV-1 near full-length amplification and bulk NGS. For subject QZ-L-06, both plasma RNA and cell-associated proviral DNA sequences at every time point were successfully amplified. The total HIV-1 DNA for LZ-L-02 at T3 and for QZ-L-06 at T4 is shown in Table S1.

**TABLE 1:** HLA class I genotype of two LTNPs in the study

	HLA-A*1	HLA-A*2	HLA-B*1	HLA-B*2	HLA-C*1	HLA-C*2
LZ-L-02	11:2	33:03	40:01	58:01	03:02	07:208
QZ-L-06	02:07	11:01	13:01	51:01	03:04	14:02

### Phylogenetic and evolutionary analyses 

#### Phylogenetic and evolutionary analyses of HIV-1 consensus sequences

**Fig 2:**
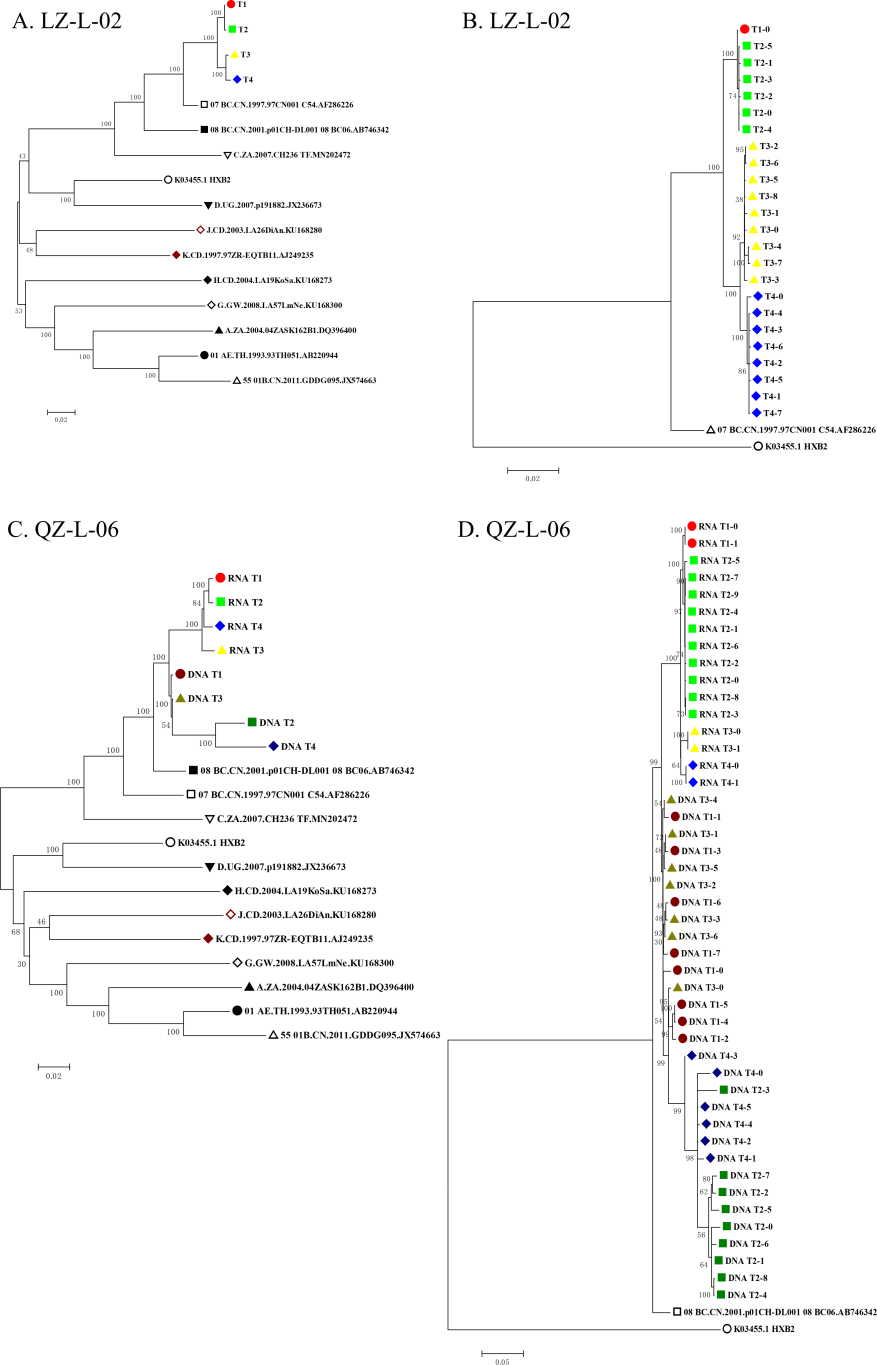
Phylogenetic analysis of HIV-1 near full-length sequences (HXB2: 1023-9024) from LZ-L-02 and QZ-L-06. (**A and C**) Consensus sequence trees display four longitudinal time points (T1–T4), with RNA sequences marked by light-colored symbols (light red circle: T1, light green square: T2, light yellow triangle: T3, and light blue diamond: T4) and DNA marked by dark-colored counterparts (dark red circle: T1, dark green square: T2, dark yellow triangle: T3, and dark blue diamond: T4). Reference sequences (black or dark red symbols) include prototype strains of subtypes A, B, C, D, G, J, K, H, and CRFs 01_AE, 07_BC, 08_BC (Los Alamos HIV Database). (**B and D**) Quasispecies trees show intra-host diversity, where distinct symbol shapes (circles: T1, squares: T2, triangles: T3, and diamonds: T4) represent viral variants of different time points under the same color scheme. Scale bars: 0.02 (**A–C**) and 0.05 (**D**) substitutions per site.

We constructed an ML phylogenetic tree of consensus sequences yielded from bulk NGS sequencing (Fig. 2). For both subjects, sequences obtained at different time points were clustered in highly supported monophyletic clusters, indicating that they were infected by a single subtype variant throughout the sampling period. For subject LZ-L-02, RNA sequences formed two close subclades containing T1, T2, T3, and T4, respectively. To further assess the genetic divergence of HIV-1 viruses in two LTNPs throughout four time points, genetic distances between sequences in each time point and the sequence in T1 were calculated (Table 2). Both RNA and DNA sequences showed increasing divergence compared with that of T1. Of note, the RNA and DNA sequences derived from subject QZ-L-06 were genetically compartmentalized in a phylogenetic tree. Linear regression analyses revealed no statistically significant difference in genetic divergence in any of the three viral sequence sets (all FDR-adjusted *P* > 0.05; Table 3), suggesting limited evolution during the follow-up.

**TABLE 2:** Genetic distance between each time point sequence and T1 sequence

	T1	T2	T3	T4
LZ-L-02 RNA	0	0.001,147	0.013,555	0.015,203
QZ-L-06 RNA	0	0.004,519	0.013,415	0.009,573
QZ-L-06 DNA	0	0.042,144	0.001,926	0.052,237

**TABLE 3:** Linear regression analysis of viral sequence sets: effect estimates, significance, and model fit

Viral sequence sets	β	β, 95% CI	*P* value	FDR adjusted *P*	R^2^
LZ-L-02 RNA	0.007	[−0.032, 0.047]	0.265	0.795	0.837
QZ-L-06 RNA	0.0025	[−0.044, 0.049]	0.617	0.879	0.321
QZ-L-06 DNA	0.005	[−0.327, 0.337]	0.879	0.879	0.036

#### Phylogenetic and evolutionary analyses of HIV-1 viral quasispecies

Haplotypes of viral quasispecies were reconstructed by CliqueSNV, and ML trees were subsequently constructed (Fig. 2B and D). Haplotypes of plasma RNA were compartmentalized by sampling time, both in the trees of LZ-L-02 and QZ-L-06. However, haplotypes of DNA of QZ-L-06 from different time points were genetically intermingled, sharing the same topological structure in ML tree of consensus sequences.

### Intra-host diversity

#### Number and frequency of intra-host single-nucleotide variants

**Fig 3:**
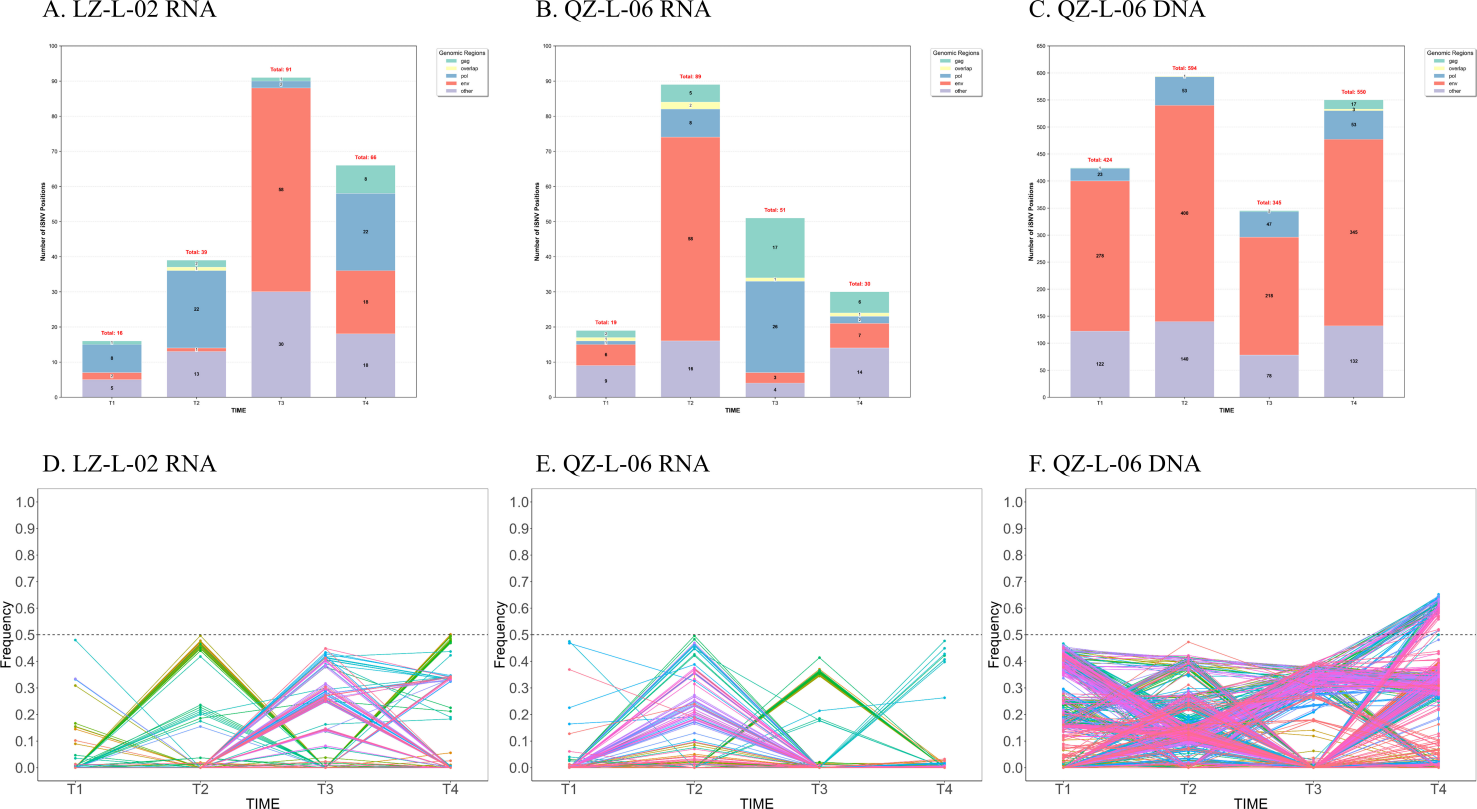
Longitudinal analysis of HIV-1 iSNVs in LZ-L-02 and QZ-L-06. (**A–C**) The number of mutation sites detected in viral sequences from longitudinal time points (T1–T4), with (**A**) RNA mutations in LZ-L-02, (**B**) RNA mutations in QZ-L-06, and (**C**) DNA mutations in QZ-L-06. The yellow overlapping region represents the coding sequence overlapped by the *gag* and *pol* genes. (**D–F**) The frequency dynamics of these mutation sites with (**D**) RNA mutations in LZ-L-02, (**E**) RNA mutations in QZ-L-06, and (**F**) DNA mutations in QZ-L-06. Each colored line represents distinct mutation profiles across the four time points. The dashed horizontal line at 0.5 frequency indicates the dominant variant threshold.

We quantified iSNVs with frequency >0.01 in at least one time point for each subject. The number and frequency of iSNVs are shown in Fig. 3. Both subjects exhibited a relatively low number of iSNVs in RNA sequences, whereas the number was 5-fold to 10-fold higher in DNA sequences.

For LZ-L-02, the frequency of iSNVs in RNA sequences remained below 0.5 at all nucleotide positions, with some positions showing an increase in frequency at T2 and T3, followed by a decline at T4 (Fig. 3D). Similarly, for RNA sequences of QZ-L-06, the frequency of iSNVs did not exceed 0.5, and most positions exhibited an increasing pattern at T2, followed by a decline. In contrast, for DNA sequences in QZ-L-06, a certain number of positions at T4 displayed a frequency over 0.5, suggesting that these mutations become dominant and may persist over time.

#### Shannon entropy and relative genetic diversity

**Fig 4:**
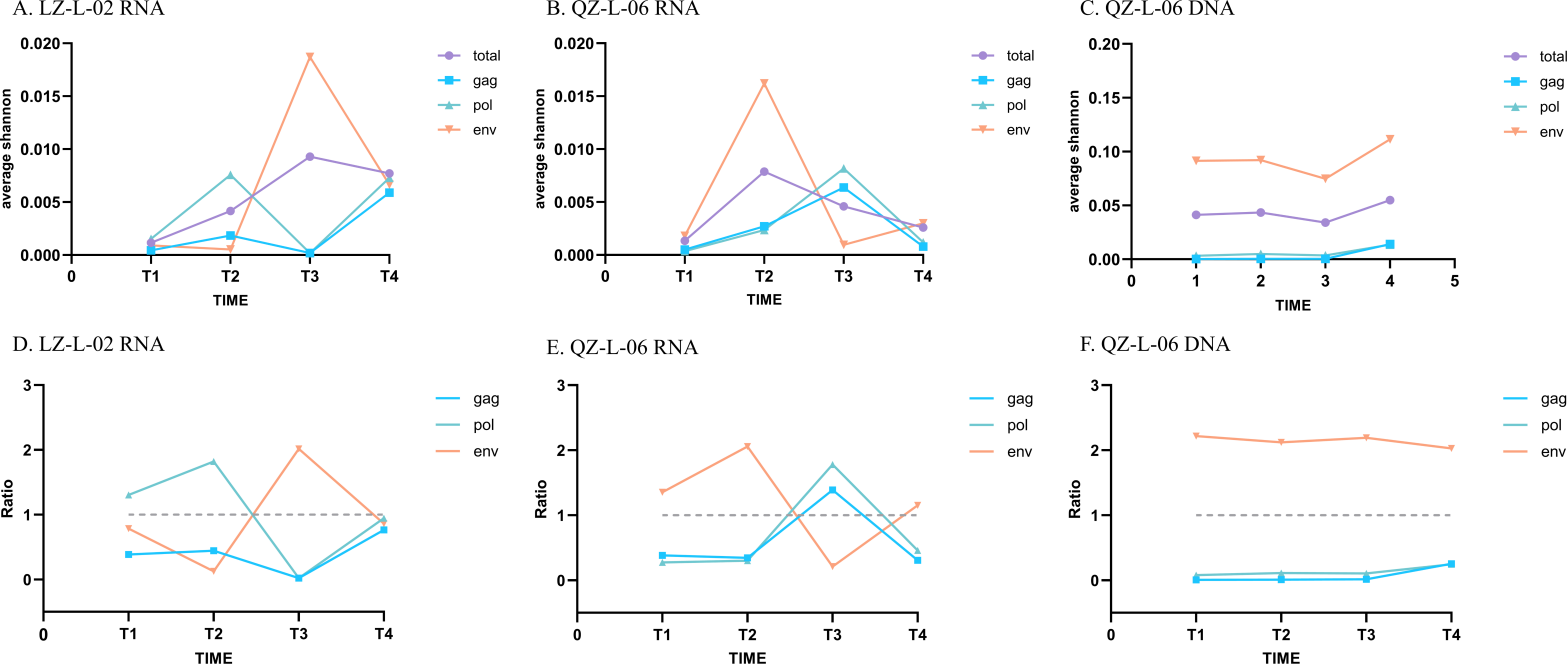
Intra-host diversity in LZ-L-02 and QZ-L-06 measured by position-wise Shannon entropy (*Hi*)*. (**A–C**) Intra-host diversity of HIV-1 viral sequences in two LTNPs. (**D–F**) Relative diversity of *gag*, *pol,* and *env* genes in two LTNPs. Blue lines: *gag* region, green lines: *pol* region, orange lines: *env* regions, and purple lines: whole genes. * The position-wise Shannon entropy (*Hi*) at position *i* was calculated as: *Hi* = −∑*_α_*_∈[_*_A_*,*_C_*,*_G_*,*_T_*_]_ *p_i_α* log_2_*p_i_α*, where *p_i_a* is the frequency of nucleotide α at position *i.* The mean Shannon entropy across a genomic region of length *N* was then computed as *avg H =* $\frac{1}{N}\sum_{i=1}^{N}Hi$，where *N* is the length of the viral sequence or gene region.

The mean Shannon entropy (*avgH*), another indicator of intra-host diversity, showed a similar pattern to that of the corresponding iSNVs (Fig. 4A through C). Consistent with the trends in iSNV numbers, the *avgH* for DNA sequences was higher than that for matched RNA sequences. We also calculated the relative diversity of *gag*, *pol,* and *env* genes (Fig. 4D through F). For RNA sequences, the relative genetic diversity of three structural genes exhibited greater fluctuations over time. By contrast, the relative diversity of all three structural genes remained stable in DNA sequences from QZ-L-06, with *env* exhibiting the highest diversity across time points.

#### Sensitivity analysis

To ensure the robustness of our analysis, we re-evaluated the frequency change trends of iSNVs and calculated the Shannon entropy after adjusting the filtering thresholds to 0.05 and 0.10, respectively (Fig. S1 to S3). This step effectively reduced potential sequencing noise while preserving the low-frequency variant spectrum of interest. The results demonstrated the robustness of our key findings; under different threshold criteria, the frequencies of iSNVs and their genetic diversity in the QZ-L-06 DNA remained consistently higher than those in its RNA counterpart, and the variation pattern of the mean Shannon entropy across time points was consistently maintained.

#### Amino acid mutation

**Fig 5:**
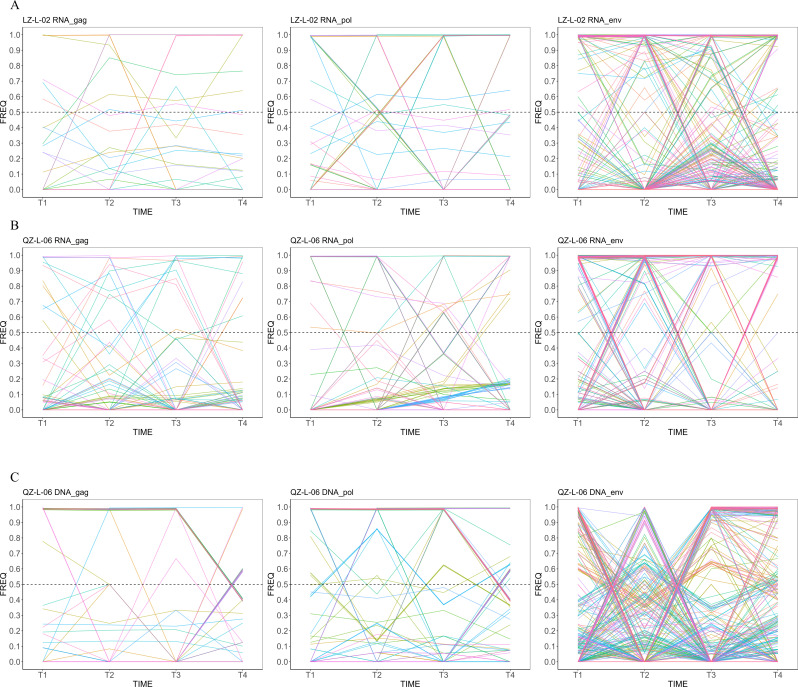
Dynamic changes of HIV-1 intra-host amino acid mutations in *gag*, *pol*, and *env* gene regions from LZ-L-02 and QZ-L-06. (**A**) Intra-host amino acid mutations of LZ-L-02 RNA. (**B**) Intra-host amino acid mutations of QZ-L-06 RNA. (**C**) Intra-host amino acid mutations of QZ-L-06 DNA. Each colored line represents distinct mutation profiles across the four time points.

The number and frequency changes of amino acid mutation over time are presented in Fig. 5 (The corresponding numerical data are presented in Supplemental material). The number of amino acid mutations is significantly lower than that of iSNVs. Both DNA and RNA sequences exhibit the highest number of mutations in the *env* gene, consistent with the number of iSNVs. The trends in mutation frequency fluctuated, often increasing sharply followed by a sudden decrease. A significant difference in the number of amino acid mutations was observed between the RNA and DNA sequences of QZ-L-06 (Fig. 5B and C). For example, mutation counts located in the *gag* gene were higher in RNA sequences than in DNA sequences, but the situation was reversed for the *env* gene.

### Characteristics of the variable region in the *env* gene

**Fig 6:**
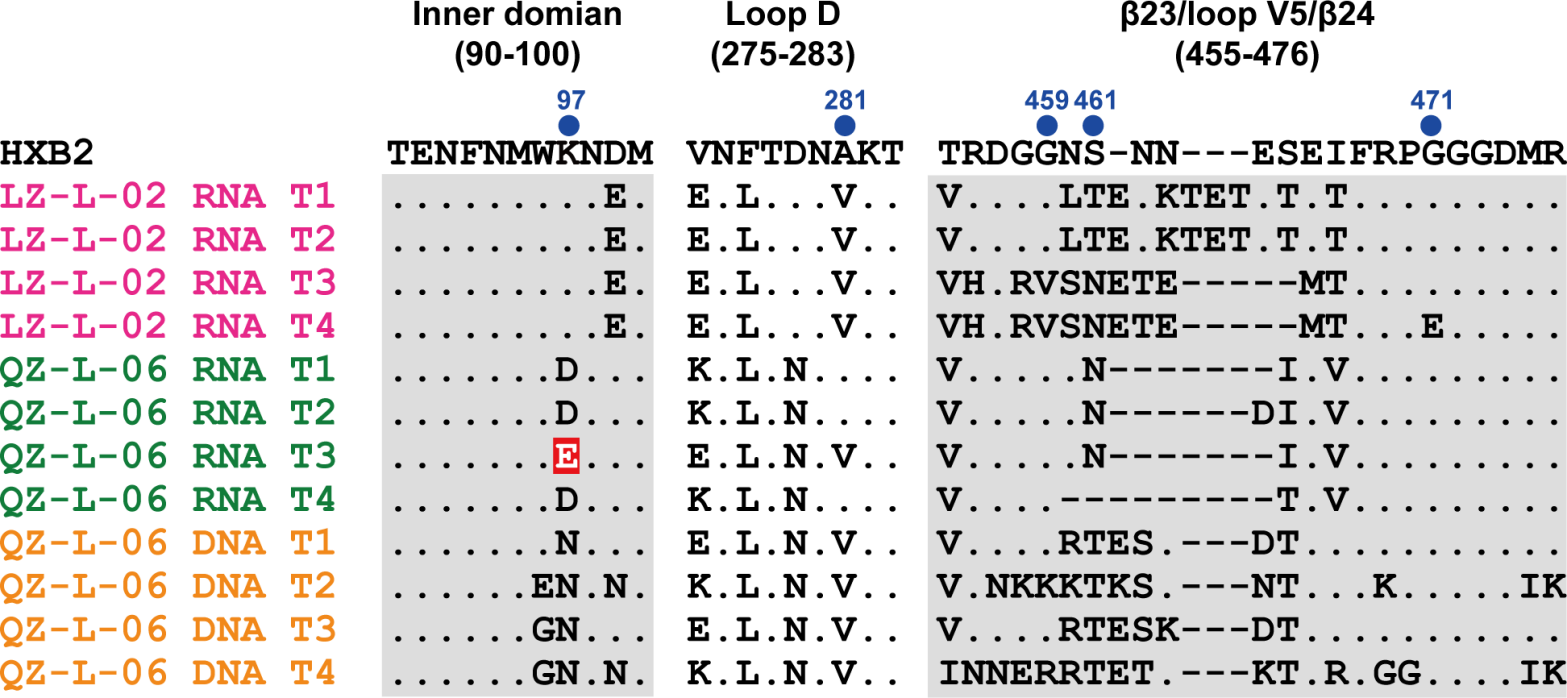
Alignment of the amino acid sequence of two LTNPs in the contact regions of CD4bs. The locations of the contact regions, including the inner domain, loop D, and β23/loop V5/β24, along the HIV-1 gp120 are indicated above the aligned sequences (HXB2 numbering is indicated). Blue dots represent signature contact residues for CD4bs bNAbs. Residues different from the HIV-1 HXB2 sequence are indicated. Dots in the alignment represent identical residues with HXB2, and dashes represent gaps introduced to preserve the alignment. Residues highlighted in red background indicate mutations associated with resistance against CD4bs bNAbs.

The features of variable regions (V1–V5) in *Env* are highly associated with broadly neutralizing antibody (bNAb) activity and immune escape. We analyzed the length of V1–V5 and the number of potential N-linked glycosylation sites (PNGS) (Table 4). In line with the aforementioned results, amino acid (AA) sequences of RNA viruses and the contemporaneous DNA viruses are discrepant. The V1 region in the two LTNPs had a moderate length of 24–31 amino acids, compared to the median length of 27 amino acids in the Los Alamos HIV database (26). Moreover, the length and number of PNGs of V1 and V3 remained stable over time. The V5 region of the two LTNPs, measured at the latest time point from the RNA virus, was shorter in length and had fewer PNGS than the HXB2 reference. For reference, the V5 region in sequences used to elicit bNAbs or in vaccine trials typically comprises 7–12 amino acids (27, 28). Amino acid variations in the inner domain, Loop D, and β23/loop V5/β24 region were analyzed, which are critical for recognition by CD4bs bNAbs. Signature substitutions associated with broadly resistant against CD4bs bNAbs (29), including K97E, A281G/L/D, G459D, N461A, and G471Q, were largely absent in both LTNPs (Fig. 6).

**TABLE 4:** Amino acid length and number of potential N-linked glycosylation sites in the variable region of the *env* gene

Sample	Time point	Region^*a*^				
		V1	V2	V3	V4	V5
HXB2		27 (2)	39 (2)	36 (1)	34 (4)	10 (1)
LZ-L-02 RNA	T1	24 (3)	43 (2)	35 (1)	28 (4)	12 (1)
	T2	24 (3)	43 (2)	35 (1)	28 (4)	12 (1)
	T3	24 (3)	43 (2)	35 (1)	34 (4)	7 (0)
	T4	24 (3)	43 (1)	35 (1)	28 (4)	7 (0)
QZ-L-06 RNA	T1	31 (4)	45 (2)	35 (1)	30 (5)	7 (0)
	T2	31 (4)	45 (2)	35 (1)	30 (6)	8 (0)
	T3	31 (4)	45 (2)	35 (1)	30 (5)	7 (0)
	T4	31 (4)	45 (2)	35 (1)	30 (6)	5 (0)
QZ-L-06 DNA	T1	26 (4)	45 (3)	35 (1)	15 (2)	11 (1)
	T2	26 (4)	45 (3)	35 (1)	28 (4)	11 (1)
	T3	26 (4)	45 (2)	35 (1)	15 (2)	11 (0)
	T4	26 (4)	45 (3)	35 (1)	15 (1)	11 (1)

^*a*^ Values outside parentheses denote the amino acid length of the respective region, and values in parentheses indicate the number of potential N-linked glycosylation sites within that region.

### CTL/CD8^+^ epitope escape mutation

According to the HLA class I alleles carried by each subject, the best-defined CTL/CD8^+^ epitopes were identified, and the numbers are 17 and 18 in LZ-L-02 and QZ-L-06, respectively. The mutations located in CTL epitopes were detected (Tables 5 to 7). According to the “CTL/CD8^+^ Epitope Variants and Escape Mutations” HIV database, IW9 is restricted by the protective allele HLA-B*58:01 and was identified in the RNA sequence of LZ-L-02. AK11 was detected in both the RNA and DNA sequences of QZ-L-06. Both IW9 and AK11 have been documented as CTL escape epitopes. For the RNA sequences of LZ-L-02, six mutations showed high (>95%) and equivalent frequency across all time points, while the rest of the two mutated epitopes showed fluctuation in frequency. In the VR10 epitopes, reversion mutations to the wild-type amino acids were observed. All the CTL escape mutations detected in RNA sequences of QZ-L-06 were present as predominance with no significant change in frequency across time points. Surprisingly, mutated epitopes were in concurrence between RNA sequences and DNA sequences. For the QZ-L-06 subject, 10 of 11 mutations found in RNA sequences are also detected in DNA sequences. Notably, the QKK9 epitope mutation was only present in RNA sequences and was not observed in DNA. In line with the result of genetic diversity, DNA sequences presented a larger number of mutations in which some minor mutations initially emerged and subsequently disappeared. In the SK10 epitopes, reversion mutations to the wild-type amino acids were also observed at T4. The RL9 epitope exhibited substantial frequency fluctuations, with two mutated variants alternating as the dominant form over time.

**TABLE 5:** The frequency of restrictive CTL/CD8^+^ epitope mutation from LZ-L-02 plasma RNA sequences

Patient	Epitope (frequency %)^*a*^						
	HLA	Position	Epitope name	T1 sequence	T2 sequence	T3 sequence	T4 sequence
LZ-L-02	B*40:01	*Gag* (92–101)	IL10	IDVRDTKEAL E93D I94V K95R	IDVRDTKEAL E93D I94V K95R	IDVRDTKEAL E93D I94V K95R	IDVRDTKEAL E93D I94V K95R
	B*58:01	*Gag* (240–249)	TW10	TSTLAEQMAW Q244A I247M G248A	TSTLAEQMAW Q244A I247M G248A	TSTLAEQMAW) Q244A I247M G248A	TSTLAEQMAW Q244A I247M G248A
	B*58:01	*Gag* (308–316)	QW9	QATQDVKNW S310T E312D	QATQDVKNW S310T E312D	QATQDVKNW S310T E312D	QATQDVKNW S310T E312D
	B*40:01	*Gag* (481–489)	KL9	EKTTPLTSL K481E E482K L483T Y484T	EKTTPLTSL K481E E482K L483T Y484T	EKTTPLTSL K481E E482K L483T Y484T	EKTTPLTSL K481E E482K L483T Y484T
	B*58:01	*Pol* (530–538)	IW9	IAMESIVIW T531A T532M	IAMESIVIW T531A T532M	IAMESIVIW T531A T532M	IAMESIVIW T531A T532M
	A*33:03	*Env* (698–707)	VR10	IFVVLSVVNR V698I A700V I704V	IFVVLSVVNR V698I A700V I704V	IFAVLSVVNR (0.74) V698I I704V IFAVLSIVNR (0.26) V698I	IFAVLSVVNR V698I I704V
	B*40:01	*Env* (805–814)	QL10	QELKKSIISL N809K A811I V812I	QELKKSIISL N809K A811I V812I	QELKKSIISL (0.46) N809K A811I V812I QELKKSTISL (0.53) N809K A811T V812I	QELKKSIISL (0.61) N809K A811I V812I QELKKSTISL (0.38) N809K A811T V812I
	A*33:03	*Env* (831–838)	ER8	ELAQRLCR V832L A836L Y387C	ELAQRLCR V832L A836L Y387C	ELAQRLCR V832L A836L Y387C	ELAQRLCR V832L A836L Y387C

^*a*^ Epitopes without annotated frequencies indicate a frequency > 0.95.

**TABLE 6:** The frequency of restrictive CTL/CD8^+^ epitope mutation from QZ-L-06 plasma RNA sequences

Patient	Epitope (frequency %)^*a*^						
	HLA	Position	Epitope name	T1 sequence	T2 sequence	T3 sequence	T4 sequence
QZ-L-06	A*11:01	*Gag* (84–91)	TK8	TLYCVHEE Q90E K91E	TLYCVHEE Q90E K91E	TLYCVHEE Q90E K91E	TLYCVHEE Q90E K91E
	A*02:07 C*03:04	*Gag* (296–304)	YL9	YVDRFFKTL Y301F	YVDRFFKTL Y301F	YVDRFFKTL Y301F	YVDRFFKTL Y301F
	A*11:01	*Gag* (349–359)	AK11	ACQGVGGPSHK G357S	ACQGVGGPSHK G357S	ACQGVGGPSHK G357S	ACQGVGGPSHK G357S
	A*11:01	*Pol* (272–281)	SK10	SVPLDKDFRK E277K G278D	SVPLDKDFRK E277K G278D	SVPLDKDFRK E277K G278D	SVPLDKDFRK E277K G278D
	B*51:01	*Pol* (283–290)	TI8	TAFTIPSV I290V	TAFTIPSV I290V	TAFTIPSV I290V	TAFTIPSV I290V
	A*11:01	*Pol* (311–321)	SK11	SPAIFQCSMTK S317C	SPAIFQCSMTK S317C	SPAIFQCSMTK S317C	SPAIFQCSMTK S317C
	B*51:01	*Pol* (448–456)	IL9	VPLTEEAEL I448V	VPLTEEAEL I448V	VPLTEEAEL I448V	VPLTEEAEL I448V
	A*11:01	*Pol* (675–683)	QKK9	HIIEQLIKK Q675H	HIIEQLIKK Q675H	HIIEQLIKK Q675H	HIIEQLIKK Q675H
	A*11:01	*Env* (199–207)	SK9	SALTQACPK V200A I201L	SALTQACPK V200A I201L	SALTQACPK V200A I201L	SALTQACPK V200A I201L
	B*51:01	*Env* (416–424)	LI9	IPCRIKQII L416I	IPCRIKQII L416I	IPCRIKQII L416I	IPCRIKQII L416I
	C*03:04	*Env* (557–565)	RL9	RAIEAQQHM L565M	RAIEAQQHM L565M	RAIEAQQHM L565M	RAIEAQQHM L565M

^*a*^ Epitopes without annotated frequencies indicate a frequency > 0.95.

**TABLE 7:** The frequency of restrictive CTL/CD8^+^ epitope mutation from QZ-L-06 proviral DNA sequences

Patient	Epitope (frequency %)^*a*^						
	HLA	Position	Epitope name	T1 sequence	T2 sequence	T3 sequence	T4 sequence
QZ-L-06	A*11:01	*Gag* (84–91)	TK8	TLYCVHEE Q90E K91E	TLYCVHEE (0.89) Q90E K91E TLDCVHEE (0.08) Y86D Q90E K91E	TLYCVHEE Q90E K91E	TLYCVHEE Q90E K91E
	C*03:04	*Gag* (296–304)	YL9	YVDRFFKTL Y301F	YVDRFFKTL Y301F	YVDRFFKTL Y301F	YVDRFFKTL Y301F
	A*11:01	*Gag* (349–359)	AK11	ACQGVGGPSHK G357S	ACQGVGGPSHK G357S	ACQGVGGPSHK G357S	ACQGVGGPSHK G357S
	A*11:01	*Pol* (272–281)	SK10	SVPLDKDFRK E277K G278D	SVPLDKDFRK E277K G278D	SVPLDKDFRK E277K G278D	SVPLDKDFKK (0.6) E277K G278D R280K SVPLDKDFRK (0.4) E277K G278D
	B*51:01	*Pol* (283–290)	TI8	TAFTIPSV I290V	TAFTIPSV I290V	TAFTIPSV I290V	TAFTIPSV I290V
	A*11:01	*Pol* (311–321)	SK11	SPAIFQCSMTK S317C	SPAIFQCSMTK S317C	SPAIFQCSMTK S317C	SPAIFQCSMTK S317C
	B*51:01	*Pol* (448–456)	IL9	VPLTEEAEL I448V	VPLTEEAEL I448V	VPLTEEAEL I448V	VPLTEEAEL I448V
	A*11:01	*Env* (199–207)	SK9	SAITQACPK V200A	SAITQACPK (0.85) V200A SALTQACPK (0.13) V200A I201L	SAITQACPK V200A	SAITQACPK V200A
	B*51:01	*Env* (416–424)	LI9	IPCRIKQII (0.95) L416I IPCKIKQII (0.05) L416I R419K	IPCRIKQII L416I	IPCRIKQII L416I	IPCRIKQII L416I
	C*03:04	*Env* (557–565)	RL9	RAIEAQQHM (0.6) L565M KAIKAQQHM (0.4) R557K E560K L565M	RAIEAQQHM (0.3) L565M KAIKAQQHM (0.7) R557K E560K L565M	RAIEAQQHM (0.66) L565M KAIKAQQHM (0.33) R557K E560K L565M	RAIEAQQHM (0.45) L565M KAIKAQQHM (0.54) R557K E560K L565M

^*a*^ Epitopes without annotated frequencies indicate a frequency > 0.95.

## DISCUSSION

Several factors, including CCR5Δ32 deletion, protective HLA alleles such as HLA-B*57 and HLA-B*27, attenuated viral strains, and strong T cell activity contribute to spontaneous control in LTNPs. Although some of these immune and genetic traits are rarely detected in the broader population, recognizing the immune and genetic traits of HIV controllers can help understand the complex interplay between genetic, immune, and virological factors. These immune and genetic traits can be mimicked or translated into therapies toward broader PLWH, ultimately achieving a functional cure. In this study, we analyzed the short-term evolutionary and quasispecies diversity of plasma HIV-1 RNA and PBMC-associated HIV-1 DNA sequences from two LTNPs over a period of approximately 2 years. Our results revealed limit intra-host evolution both in plasma RNA and proviral DNA, and a higher diversity of DNA quasispecies than that of corresponding RNA quasispecies. In addition, both LTNPs harbored an appreciable number of CTL mutations, maintaining a sustainable frequency over time.

Previous studies have reported that longitudinal HIV-1 sequences from ECs are typically tightly clustered in the setting of <2 years sampling interval, but more pronounced evolutionary changes are generally observed when the sampling interval exceeds 3–4 years (30). In our study, the sampling intervals of the two LTNPs were relatively short, which were 21 months for LZ-L-02 and 16 months for QZ-L-06, respectively. The genetic divergence showed a slight increase over time in RNA sequences of two subjects, with 1.5% and 0.9% variations during sampling intervals. These findings suggest that plasma viral evolution may occur at an extremely slow rate, with minimal detectable evidence of evolution. The high level of viral replication of LTNPs might explain the evolution observed in the present study. Moreover, proviral DNA sequences of QZ-L-06 collected at different time points were highly intermingled in the phylogenetic tree and showed a progressive increase (> 5%) in genetic distance over time, which is much higher than the variability of <1% over 8 years in elite controllers (6). Proviruses within the reservoir of LTNPs often remain latent for prolonged periods, leading to insufficient replenishment of plasma virus from peripheral compartments (31, 32). This may explain why DNA continues to evolve, whereas RNA does not. Multiple studies based on single-gene amplification have reported evidence of evolutionary divergence between HIV-1 RNA and DNA sequences in ECs (30, 33, 34), which was also confirmed in our study, suggesting that PBMCs may not serve as the direct source of plasma virus. A combined analysis of DNA sequences from different anatomical compartments and plasma RNA in LTNPs will contribute to a deeper understanding of the viral origin.

We evaluated the temporal trends of intra-host quasispecies variation in two LTNPs by several indices, most of which indicate a higher diversity of circulating DNA reservoir than plasma RNA quasispecies. Substantial low-frequency mutations in DNA sequences contributed to the higher diversity. We further examined the intra-host genetic variation of the *gag*, *pol*, and *env* genes, as well as the relative genetic diversity among these genes. The *gag* gene exhibited the lowest number of iSNVs, whereas the *env* region had the highest. This can be explained that the *gag* gene has a conserved and stable structure (35), whereas the *env* gene is a key target for HIV-1 genetic variation and immune escape (36) and harbors a higher number of mutations and greater genetic diversity (37, 38). However, the relative genetic diversity patterns observed in RNA sequences were inconsistent with previous literature reports, showing substantial fluctuations across the three coding gene regions. This may be due to poor retention of viral RNA nucleotide mutations. Proviruses represent the integrated viral genome, which accumulates more mutations and remains relatively stable. In contrast, RNA mutations are predominantly transient, either failing to be transmitted through viral replication cycles or being rapidly cleared by host immunity. This dynamic equilibrium results in pronounced fluctuations in observed genetic diversity within RNA. These findings further underscore the distinct evolutionary patterns between RNA and DNA in LTNPs (39).

It is generally accepted that HIV can escape the neutralization of NAbs through mutation or glycosylation in the *env* gene. The loop length and the number of PNGs in the variable region are inversely correlated with neutralizing activity (28). The moderate length of the V1 region observed in plasma viruses of both LTNPs suggested that they did not develop immune escape from V3-glycan targeting bNAbs. Moreover, the shorter V5 region, fewer PNGS, and the absence of variation conferring broad resistance indicated that plasma viruses were sensitive to CD4bs bNAbs. Collectively, the sequence features of the variable region suggested that antibody neutralization activity is associated with the maintenance of viral control in the LTNPs in our study.

Previous studies observed a large amount of CTL escape mutations in plasma RNA or proviral DNA in spontaneous HIV-1 controllers carrying protective HLA class I alleles (17, 24). In the present study, most of the mutations in CTL epitopes exhibited high frequency and minimal change in frequency across the follow-up. Furthermore, the type and frequency of escape mutations between RNA sequence and DNA sequence were consistent, despite their phylogenetic divergence. This suggested that the mutated CTL epitopes may occur prior to sequence segregation and did not evolve during chronic infection. Given that LTNPs reach viral control soon after acute infection (40), and such large numbers of mutations are less likely to be acquired from the donors with the same HLA allele type, we tend to presume that the escape mutations were the consequence of strong selection pressure exerted by an efficient CTL response in acute infection. Nevertheless, the fixation of escape mutations indicated that the LTNPs have achieved a balance between immune response and viral replication. The relatively high viral load in the analyzed LTNPs may reflect the attenuated CD8 T cell response. How LTNPs achieve viral control despite multiple escape mutations warrants further study.

This study, being descriptive and exploratory in nature, is subject to certain limitations. First, due to the challenges of recruiting and maintaining LTNPs for longitudinal research, only two LTNPs were included. Second, the sampling period was relatively short; hence, our findings should be confirmed over a longer duration. Moreover, while our calculated template input reduces the risk of resampling bias, it does not guarantee complete capture of reservoir diversity. Finally, the analyzed proviruses include defective variants.

In summary, we deeply characterized the near-full-length genome dynamics of two LTNPs using bulk NGS. The features observed in these LTNPs include protective HLA alleles, limited viral evolution, divergence between plasma virus and PBMC-derived provirus, shorter loops in the CD4 binding sites, and a high proportion of CTL epitope mutations. Although it is unlikely that all these features are simultaneously present in a broader population of people living with HIV, they provide a valuable model for understanding the state of non-progression and potential pathways to achieve it. This suggests that a functional cure may be attainable through multiple strategies. Further studies are needed to elucidate the origin of the plasma virus and explain why the observed immune escape mutations do not confer increased pathogenicity.

## Data Availability

The raw sequencing reads generated in this study have been deposited in the NCBI Sequence Read Archive (SRA) under BioProject accession number PRJNA1371142. The assembled RNA and DNA consensus sequences for the two LTNPs are available in GenBank under accession numbers PZ011002 to PZ011013.

## SUPPLEMENTAL MATERIAL

Data S1 spectrum.02227-25-s0001.xlsx The corresponding numerical data for Fig. 5.

Fig. S1 spectrum.02227-25-s0002.tif Sensitivity analysis of iSNVs frequency change trends from LZ-L-02 and QZ-L-06.

Fig. S2 spectrum.02227-25-s0003.tif Sensitivity analysis of mean Shannon entropy from LZ-L-02 and QZ-L-06.

Fig. S3 spectrum.02227-25-s0004.tif Sensitivity analysis of relative Shannon entropy from LZ-L-02 and QZ-L-06.

Supplemental material spectrum.02227-25-s0005.docx Legends for all supplemental figures.

Table S1 spectrum.02227-25-s0006.docx Results of total HIV DNA from 2 LTNPs.

ASM does not own the copyrights to Supplemental Material that may be linked to, or accessed through, an article. The authors have granted ASM a non-exclusive, world-wide license to publish the Supplemental Material files. Please contact the corresponding author directly for reuse.
