# Supplementary figures and images for: Analysis of temporal virus evolution and intra-host diversity in long-term non-progressors by bulk next-generation sequencing
Source: Microbiol Spectr. 2026 Mar 30;14(5):e02227-25. doi: 10.1128/spectrum.02227-25 (PMC13141976; doi:10.1128/spectrum.02227-25)

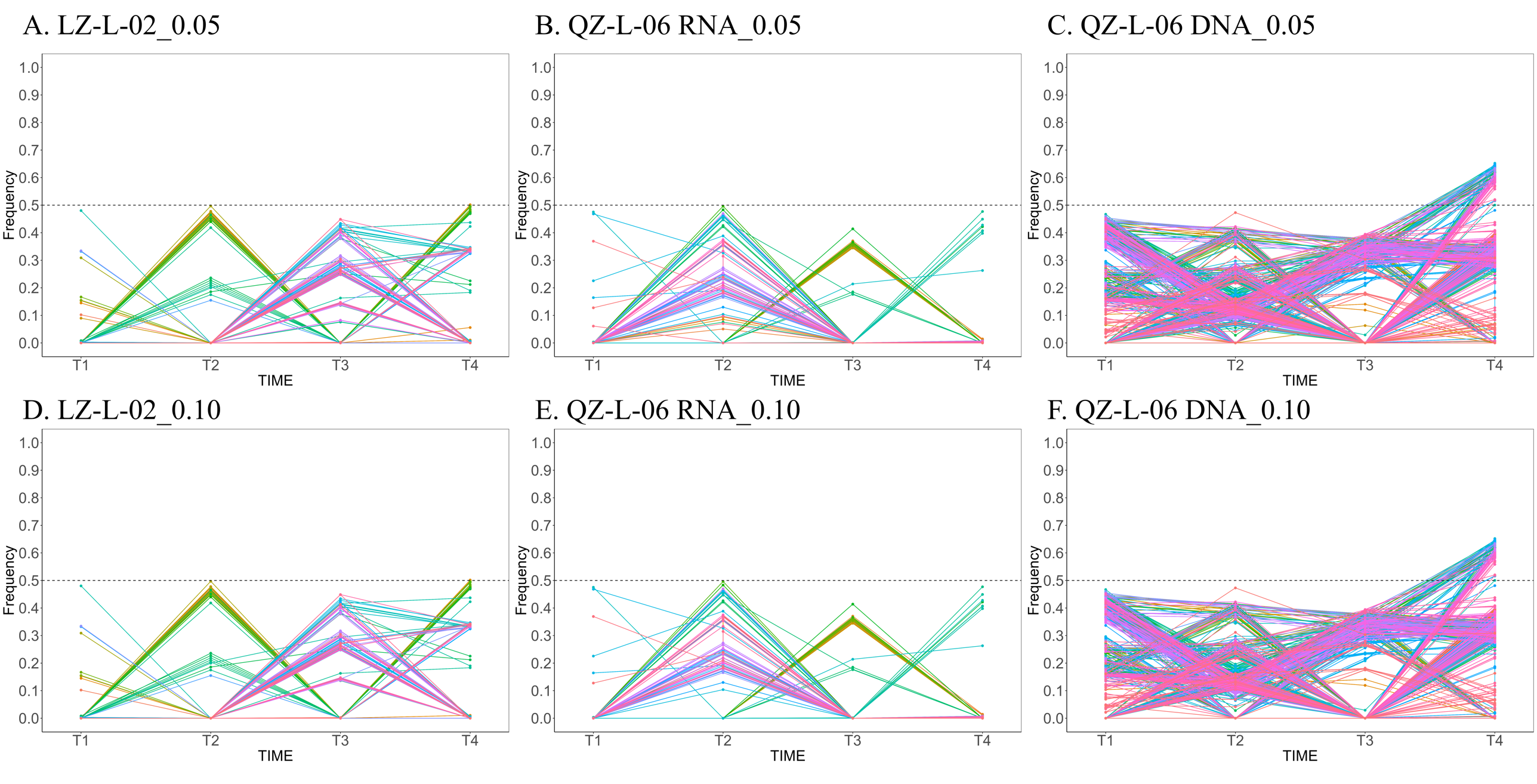

Supplement: Fig. S1 — Sensitivity analysis of iSNVs frequency change trends from LZ-L-02 and QZ-L-06. [file spectrum.02227-25-s0002.tif]

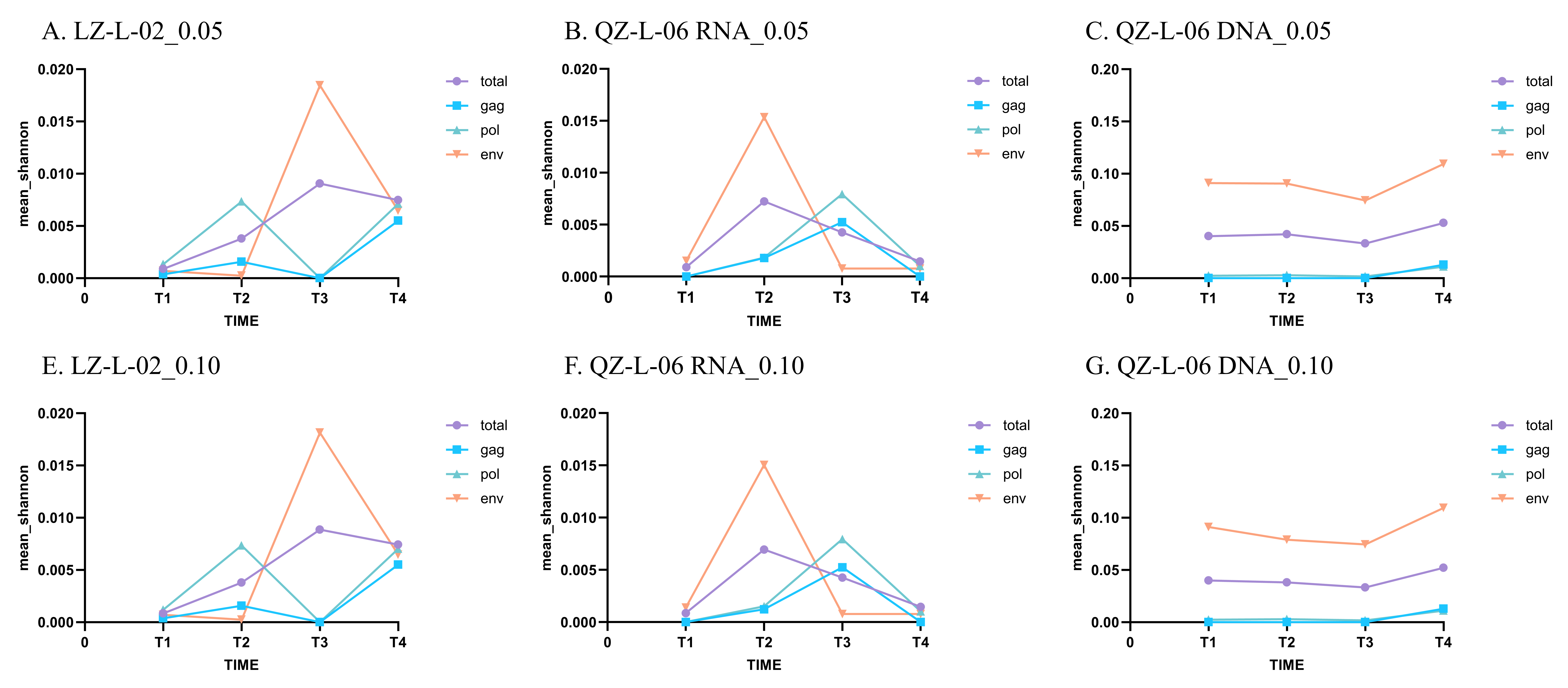

Supplement: Fig. S2 — Sensitivity analysis of mean Shannon entropy from LZ-L-02 and QZ-L-06. [file spectrum.02227-25-s0003.tif]

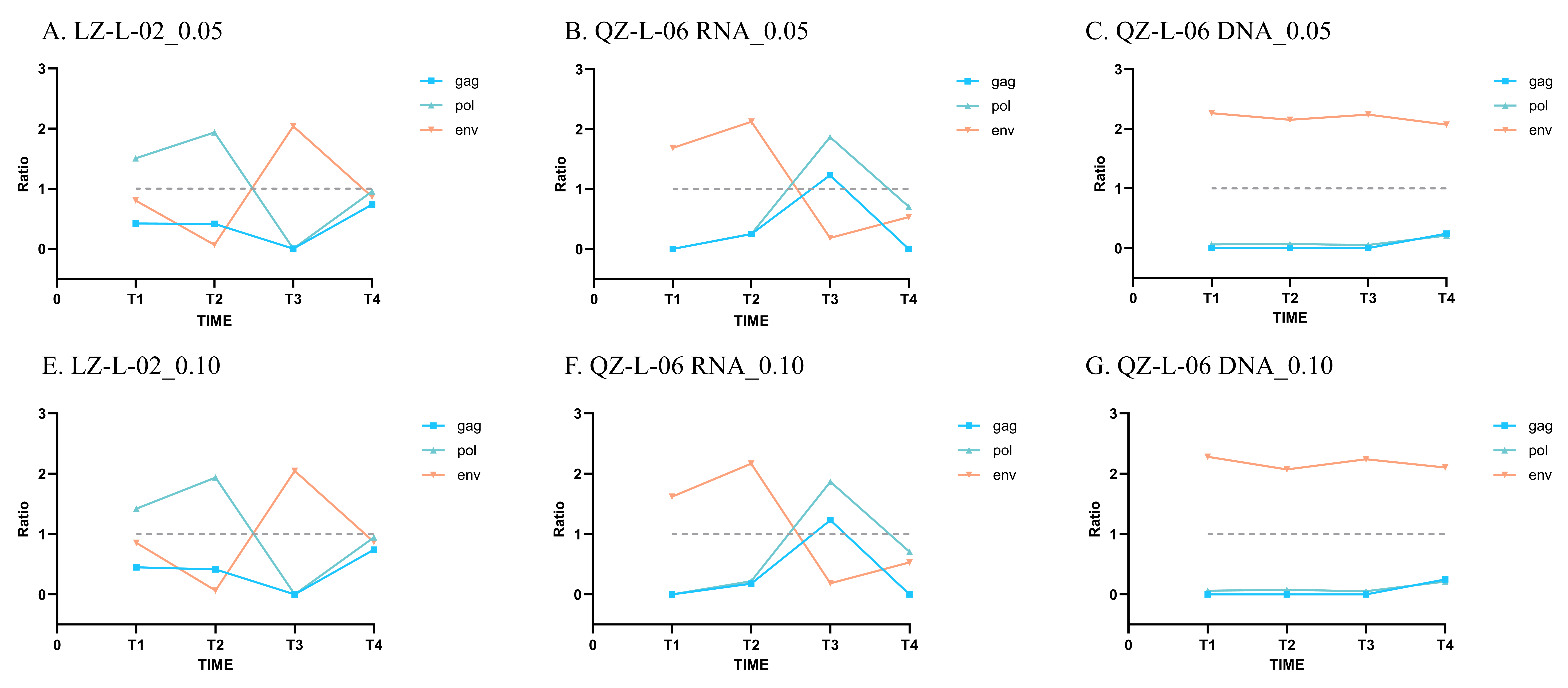

Supplement: Fig. S3 — Sensitivity analysis of relative Shannon entropy from LZ-L-02 and QZ-L-06. [file spectrum.02227-25-s0004.tif]
