# Supplementary material for: Analysis of temporal virus evolution and intra-host diversity in long-term non-progressors by bulk next-generation sequencing
Source: Microbiol Spectr. 2026 Mar 30;14(5):e02227-25. doi: 10.1128/spectrum.02227-25 (PMC13141976; doi:10.1128/spectrum.02227-25)
Supplement: Supplemental material — Legends for all supplemental figures. [file spectrum.02227-25-s0005.docx]

Supplementary Figure 1. Sensitivity analysis of iSNVs frequency change trends from LZ-L-02 and QZ-L-06. (A-C) Analysis with a 0.05 filtering threshold. (D-F) Analysis with a 0.10 filtering threshold.

Supplementary Figure 2. Sensitivity analysis of mean Shannon entropy from LZ-L-02 and QZ-L-06. (A-C) Analysis with a 0.05 filtering threshold. (D-F) Analysis with a 0.10 filtering threshold.

Supplementary Figure 3. Sensitivity analysis of relative Shannon entropy from LZ-L-02 and QZ-L-06. (A-C) Analysis with a 0.05 filtering threshold. (D-F) Analysis with a 0.10 filtering threshold.
