# Supplementary material for: Analysis of temporal virus evolution and intra-host diversity in long-term non-progressors by bulk next-generation sequencing
Source: Microbiol Spectr. 2026 Mar 30;14(5):e02227-25. doi: 10.1128/spectrum.02227-25 (PMC13141976; doi:10.1128/spectrum.02227-25)
Supplement: Table S1 — Results of total HIV DNA from 2 LTNPs. [file spectrum.02227-25-s0006.docx]

Supplementary Table S1 Total HIV-1 DNA levels of LTNPs

| Sample | Time point | total HIV DNA (copies/million PBMCs) |
| --- | --- | --- |
| LZ-L-02 | T3 | 2178 |
| QZ-L-06 | T4 | 509 |
